# Are we on the same wavelength? Supervisor-subordinate cognitive style congruence and its association with supervisors’ self-awareness through leader member exchange

**DOI:** 10.3389/fpsyg.2025.1583837

**Published:** 2025-06-18

**Authors:** Faisal AlReshaid, Alper Erturk, Razan Alkhayyat, Farid Abdallah, Oualid Abidi, Marcelle De La Roche

**Affiliations:** ^1^College of Business and Economics, American University of Kuwait, Safat, Kuwait; ^2^College of Business, Australian University, West Mishref, Kuwait; ^3^Independent Researcher, Istanbul, Türkiye

**Keywords:** transformational leadership, self-awareness, cognitive styles, leader-member exchange, polynomial analysis

## Abstract

**Introduction:**

Leaders’ self-awareness is essential to leadership effectiveness. Cognitive styles—how individuals perceive and process information—are key factors in fostering self-awareness. Drawing on Social Identity Theory (SIT), this study explores whether cognitive style congruence between supervisors and subordinates enhances supervisors’ self-awareness, and whether Leader-Member Exchange (LMX) mediates this relationship.

**Methods:**

We used a time-lagged dyadic design and multilevel polynomial regression to analyze data from 189 subordinates and 36 supervisors. Cognitive styles were measured across three dimensions: knowing, planning, and creating. We assessed congruence between supervisors and subordinates and tested LMX as a mediator of its relationship with supervisors’ self-awareness in the context of transformational leadership.

**Results:**

Findings show that LMX fully mediates the relationship between cognitive style congruence (for knowing and creating styles) and supervisors’ self-awareness. Additionally, LMX is highest when supervisors and subordinates share high congruence in these styles. No significant effects were found for the planning style.

**Discussion:**

The study highlights the value of aligning cognitive styles in leader–follower pairs. High cognitive congruence enhances LMX quality, which in turn fosters leader self-awareness—an important precursor to transformational leadership effectiveness.

## 1 Introduction

Over the past decades, leadership has not been explained as an individual trait, yet rather is presented as shared, dyadic and interactional complex social dynamic (Avolio, 2007; Avolio et al., 2009). There has been a wide agreement among scholars that leadership is mutually established by leaders and their followers. Thus, it would be a more suitable approach for leadership models to take into account followers, their roles, cognitions and perceptions (Castro et al., 2008).

Self-ratings alone are not considered good predictors of how well an individual is performing (Atwater and Yammarino, 1997; Fleenor et al., 2010). Thus, the concept of managerial self-awareness defined as the degree of agreement of leaders and their followers’ perception on leaders’ behaviors has attracted significant attention (self-other agreement - SOA). Managerial self-awareness is related to positive individual and organizational level work-related outcomes, such as higher employee job satisfaction and effective leadership (Atwater and Yammarino, 1997; Church, 1997; Tekleab et al., 2008). Thus, enhancing self-awareness is considered crucial for effective leadership, since perceptual disagreement between leaders and their followers may indicate leaders’ incompetency to communicate or failure to take action according to the demands of followers. As Lee and Carpenter (2018, p. 253) clearly emphasize, “agreement between a leader’s self-rating of leadership and ratings from the leader’s subordinates, peers, and superiors (i.e., self-other agreement) is critical to understanding leadership,” but surprisingly very little is known about the factors shaping self-other agreement.

Research shows that differences in the leaders’ and subordinates’ preferences on organizing and processing information or experience may lead to differentiations in their perceptions (Allinson et al., 2001). This idea evokes that the cognitive styles, defined as the way how people perceive information and how they use it to guide their attitudes and behaviors (Cools and Van den Broeck, 2007), may be relevant and serve as a possible antecedent of managerial self-awareness. Previous research demonstrates that people having different cognitive styles may have dissimilar perceptions regarding work-related attitudes and behaviors (Chilton et al., 2005; Cools et al., 2009). Therefore, supervisors and their subordinates’ cognitive styles congruence may have a positive influence on supervisors’ self-awareness.

Having cognitive style congruence, leaders may establish effective interactions and exchanges with their subordinates and are expected to have higher self-awareness based on the high-quality exchanges with their subordinates (Allinson et al., 2001). However, how those processes may affect self-awareness have not sufficiently been addressed in the leadership literature (Avolio et al., 2004; Castro et al., 2008; Cools et al., 2009). Thus, there is clearly a need for further research to achieve a better insight of the intervening mechanisms through which cognitive congruence influences leaders’ self-awareness. Hence, to address this gap, this study examines the underlying process through which congruence between supervisors’ and subordinates’ cognitive styles influences leaders’ self-awareness on their own leadership behaviors by exploring the mediating role of social interactions between leaders and employees on the relationship between cognitive style congruence and self-awareness.

From theoretical perspective, Social Identity Theory (SIT) offers insights into how cognitive style congruence between supervisors and subordinates can influence supervisors’ self-awareness by shaping their social identity within the organizational context. SIT theory suggests that individuals derive a significant part of their self-concept from the groups they belong to, including their work group or organizational unit (Albert et al., 2000; Hogg and Terry, 2000). It proposes that people strive to maintain a positive social identity by favorably comparing their group (in-group) with others (out-groups). When supervisors and subordinates share similar cognitive styles, they are more likely to perceive themselves as part of the same social identity within the organization. SIT posits that individuals’ self-concept and self-awareness are influenced by their group memberships and social identity. In the context of supervisor-subordinate relationships, a strong social identity between them (facilitated by cognitive style congruence) could enhance the supervisor’s self-awareness through enhanced relationship and rapport between them.

Our study is expected to contribute to literature in several ways. First, while there are attempts to investigate the possible consequences of managerial self-awareness (e.g., Atwater et al., 2005; Sosik, 2001; Sosik and Godshalk, 2004), there are only a few studies on the antecedents of self-awareness (e.g., Atwater et al., 2009). Though it seems plausible to suggest that the differentiations in information-processing styles between leaders and employees may influence the leaders’ self-awareness, according to our knowledge and investigation, no research to date has investigated the potential direct and indirect influence of cognitive congruence on the leaders’ self-awareness empirically. Thus, this study aims to close this gap in the literature and addresses the call for research to investigate different antecedents of managerial self-awareness (Atwater et al., 2009; Vecchio and Anderson, 2009) by empirically exploring the potential influence of cognitive congruence between supervisors and subordinates on supervisors’ self-awareness. Second, despite the fact that researchers have mentioned the importance of self-awareness on decision making processes, they also criticized self-awareness theory for not considering contextual variables, which are important for decision making process (Atwater et al., 2009). Hence, our examination of cognitive style fit as antecedent to managerial self-awareness addresses this limitation. Third, having leader member exchange as a mediator in proposed research model, our study also aims to respond to calls for further studies on the integration of leader member exchange with more variables to explore its further possible influence on employees and their perceptions (Erdogan and Bauer, 2014; Martin et al., 2016; Nahrgang and Seo, 2016). Finally, although a large emphasis has been put on the importance of cognitive congruence in theoretical studies, only a few research has empirically examined whether cognitive (mis)congruence actually leads to expected consequences. Hence, by following a contingency perspective, in which the relationship between the cognitive congruence and the leadership rating congruence through the mediation of LMX, this study addresses the call for research incorporating cognitive congruence especially in terms of dyadic leader-subordinate fit into leadership studies (Cools et al., 2009).

**Figure 1:**
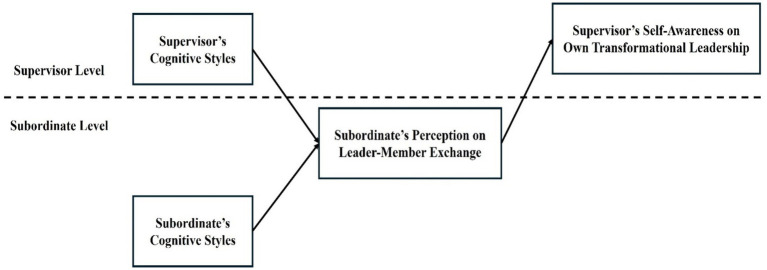
The conceptual model of the study.

As a summary, in this study, through the theoretical perspective of Social Identity Theory (SIT), we suggest that cognitive style congruence between supervisors and their subordinates would have a positive relationship with supervisors’ self-awareness on their own transformational leadership, and leader-member exchange (LMX) would mediate that relationship. The conceptual model of this study is depicted in Figure 1.

## 2 Literature review and hypotheses development

### 2.1 Social identity theory

Social Identity Theory (SIT) provides a robust framework for understanding how supervisor-subordinate relationships are influenced by cognitive style congruence and how this, in turn, impacts supervisors’ self-awareness within organizational contexts. According to SIT, individuals derive a significant part of their self-concept from the groups to which they belong, termed as social identities (Ashforth and Mael, 1989). In the workplace, supervisors and subordinates form distinct social identities based on their roles and interactions, which are influenced by shared perceptions and commonalities, such as cognitive styles (Albert et al., 2000).

Cognitive style congruence refers to the degree to which supervisors and subordinates share similar ways of perceiving and processing information (Allinson et al., 2001). When there is congruence in cognitive styles between supervisors and subordinates, it enhances their mutual understanding and communication, fostering a sense of in-group identification (Hogg and Terry, 2000). This alignment in cognitive styles facilitates the development of high-quality relationships characterized by trust, cooperation, and shared goals.

The quality of these relationships, influenced by cognitive style congruence, plays a crucial role in shaping supervisors’ self-awareness. Supervisors in high-quality exchange relationships with congruent subordinates are more likely to receive accurate and constructive feedback about their leadership behaviors (Ashford and Cummings, 1983). This feedback mechanism is instrumental in enhancing supervisors’ self-awareness by providing insights into how their actions and decisions are perceived and interpreted by others within their in-group (Yukl, 2020). Moreover, supervisors who share cognitive style congruence with their subordinates may experience enhanced self-perceptions and a clearer understanding of their strengths and weaknesses as leaders (Haslam et al., 2015).

In conclusion, Social Identity Theory illuminates how supervisor-subordinate cognitive style congruence influences supervisors’ self-awareness through the development of in-group dynamics and the quality of exchange relationships. This theory underscores the importance of shared perceptions and mutual understanding in fostering effective leadership within organizations. By exploring these dynamics, organizations can cultivate environments that promote not only cognitive diversity but also interpersonal cohesion, ultimately contributing to enhanced leadership effectiveness and self-awareness among supervisors.

### 2.2 Supervisor self awareness

Self-awareness, within an organizational context, is defined as “the ability to reflect on and accurately assess one’s own behaviors and skills as they are manifested in workplace interactions” (Church, 1997, pp. 281). Self-awareness is considered an important capability for leader effectiveness and is established by way of the consistency between the leader’s view of his/her own leadership and the image he or she creates on followers, so that they can work together in order to create a substantial work-related performance (Berson and Sosik, 2007; Sosik, 2001). In addition, self-awareness is also acknowledged as a fundamental skill that leaders need to obtain to enhance their efficacy (Berson and Sosik, 2007). To maintain and foster their effectiveness, leaders need to have sufficient awareness on their leadership behaviors and how those behaviors are perceived and interpreted by the followers.

Self-other agreement indicates a mutual understanding between a leader and followers and helps them customize their behavior in accordance with the needs of each other.

Researchers have suggested that greater self-awareness is a significant aspect for individual development and can stimulate leaders to empower themselves and their employees and thus is considered critical for leader effectiveness (Caldwell and Hayes, 2016; Lee and Carpenter, 2018). Previous research has also widely demonstrated that self-awareness has important implications, such as improved performance (Atwater and Yammarino, 1992; Atwater et al., 2005); effective leadership (Atwater et al., 1998); successful mentoring (Sosik and Godshalk, 2004), and positive work-related attitudes (Sosik, 2001).

Researchers have also conceptualized self-awareness as a sub-component of authentic leadership (Gardner et al., 2011; Walumbwa et al., 2008). Thus, from authentic leadership perspective, several researchers have demonstrated the positive link from self-awareness to various work-related attitudes and behaviors; such as helping behavior, employee voice behavior, risk perception, work engagement and organizational commitment (e.g., Hirst et al., 2016; Nielsen et al., 2013; Wang et al., 2014). Banks and his colleagues’ recent meta-analysis (Banks et al., 2016) has also demonstrated that self-awareness as a factor of authentic leadership is significantly and positively associated with LMX, job satisfaction, mutual trust and job engagement.

However, only a few studies focus on the antecedents of self-awareness, which provides inadequate understanding regarding the predictors, such as gender, age, education, personality, organizational type and functional area (Atwater et al., 2009; Ostroff et al., 2004; Smither et al., 2005). Yet, an important and critical factor that is basically ignored is the individuals’ information processing styles that define how individuals perceive and interpret the other’s behaviors. Thus, our main focus is to investigate the direct and indirect influence of cognitive style congruence between the leaders and their followers on the leaders’ self-awareness through LMX.

On the other hand, research shows that transformational leadership is the emerging leadership style that has a positive impact on organizational and leadership effectiveness in Turkish business context (Akdere et al., 2021; Erkutlu, 2008; Erturk et al., 2018). In countries such as Turkiye, which is characterized by highly collectivist organizational culture emphasizing the central role trust, employees are expected to communicate their opinions and suggestions to their supervisors regularly, and feel comfortable sharing those ideas through reliable and trustworthy communication (Aycan et al., 2000; Erturk, 2008). In such contexts, communication between leaders and employees generally involves having followers’ input, as well as striving for information and feedback from them. Therefore, leaders and managers in Turkish organizations most likely have a greater likelihood of knowing their followers’ expectations, perceptions and opinions with a high degree of accuracy (i.e., self-awareness) due to the open and candid communication encouraged in these contexts (Erturk et al., 2018; Karakitapoğlu-Aygün et al., 2021).

The current study examines self-awareness utilizing transformational leadership. Recent research defines transformational leadership as a construct through which leaders induce and encourage followers to make self-sacrifices, dedicate to challenging objectives, and perform beyond expectations (Piccolo et al., 2012). Recent meta analyses also yield that transformational leadership currently seems to be the most effective and dominant concept in leadership research (Gottfredson and Aguinis, 2017). Research reveals that, since transformational leadership is a relation-oriented style, congruence between leaders’ and followers’ assessments on leader becomes much more critical for the effectiveness of the leader (Lee and Carpenter, 2018; Sosik and Megerian, 1999). Since transformational leaders concentrate on influencing and inspiring followers to share and pursue a common understanding of the leader’s vision and goals (Tekleab et al., 2008), self-awareness defined as self-other agreement on leadership is therefore very important for transformational leaders to obtain a shared understanding and common ground throughout the organization. Transformational leaders having a significant degree of self-awareness are likely considered more effective and successful by their subordinates than those having a low level of self- awareness (Sosik and Megerian, 1999; Tekleab et al., 2008).

### 2.3 Cognitive style congruence

Cognitive styles are generally defined in the literature as the information-processing habits, in which individuals typically and consistently process information, reach conclusions or judgments based on their observations and respond to their environment (Cools et al., 2009; Erdil and Tanova, 2015). How people process information and use in decision making is not only important from a leadership perspective, but also important from marketing, entrepreneurship, and communication, especially in today’s era dominated by the strategies toward globalization and digitalization (e.g., Al-Kenane et al., 2025; Alkharafi and Alsabah, 2025; Moharrak et al., 2025; El-Dabt et al., 2025). That is why studying cognitive styles is very crucial and might bring new perspectives for leaders, who need to make solid and rational decisions in today’s ambiguous business environment.

In our study, to conceptualize cognitive style (mis) congruence, we utilize Cognitive Style Indicator (CoSI) scale, which has been developed and validated by Cools and Van den Broeck (2007). CoSI has three different cognitive styles, namely knowing, planning and creating styles. Individuals with a high knowing style are characterized by a preference for facts and a logical and rational way of processing information by looking for data in detail. Individuals having high planning style show a preference for objective, structured and conventional approach, and attribute significant importance to organization, preparation and planning in order to reach goals. Individuals having high creating style like uncertainty and freedom, consider difficulties as opportunities, and have an inclination for creative and flexible way of decision making.

### 2.4 Cognitive style congruence and LMX

Social exchange theory has been proposed to explore associations between the leaders and followers and in this study is operationalized as leader-member exchange (hereafter, LMX) (Wayne et al., 1997). LMX is expressed as a sign of the quality of an employee’s social exchange with his/her leader (Graen and Uhl-Bien, 1995). Thus, LMX interactions encompass the exchange of information and mutual emotions, such as trust, respect, and loyalty (Wayne et al., 1997).

Recent studies have suggested that implicit leadership theories play an important role in LMX process (Epitropaki and Martin, 2005; Foti et al., 2017; Riggs and Porter, 2017; Tsai et al., 2017). Researchers also state the significance of cognitive congruence in the context of HR processes, such as recruitment, selection/placement and HR planning (Armstrong and Sadler-Smith, 2006). On the other hand, cognitive miscongruence has also shown to be related to decreased performance, increased stress and turnover intentions (Brigham et al., 2007; Chilton et al., 2005). Research also suggests that individual differences or congruence in cognitive styles influence the environment formed by interpersonal interactions and performance (Allinson et al., 2001; Armstrong et al., 2004; Vanderheyden and De Baets, 2015).

Cognitive similarity promotes effective communication and mutual fondness between people (Armstrong et al., 2002; Vanderheyden and De Baets, 2015). Erdil and Tanova (2015) suggest that high level of cognitive congruence increases the employees’ communication satisfaction with their supervisors. In a recent research, for example, Emirza and Katrinli (2022) shows the significant relationship between leader-follower similarity in construal level of the work and leader-member exchange quality. In another resent study, Van Beurden et al. (2025) found out that relationship quality between employees and their managers, as well as employees’ commitment and performance, are affected positively when employees perceptions regarding the work practices align at high levels with those of their managers. Thus, cognitive congruence may create a positive impression in the mind of the leader, and in turn increases both the quality of a dyadic relationship and the employees’ liking of their leader (Armstrong et al., 2004). Hence;

*H1:* Congruence on the cognitive styles of the leaders and their subordinates will be positively related to subordinates’ perception on LMX with their leaders.

### 2.5 Mediating role of LMX between cognitive style congruence and self-awareness

High quality social exchanges are characterized by frequent exchange of valued resources and engagement in activities beyond formal required employment relationship (Liden and Maslyn, 1998). Over time, these high-quality exchange relationships turn into social relations that influence employees’ interpretations and perceptions regarding their leaders (Wang et al., 2005). High levels of LMX indicates a mutual understanding between a leader and his/her subordinates that helps them adapt their behavior in accordance with the needs and demands of each other and the organization and increase positive work-related outcomes (Dulebohn et al., 2012; Martin et al., 2016; Sui et al., 2016). Supervisors having a high level of LMX with their subordinates are able to better monitor their own behaviors and their subordinates’ perceptions and interpretation on their behaviors, have the ability to grasp various standpoints of subordinates and thus they can obtain a high self-awareness (Sosik et al., 2002). Hence, supervisors who have self-awareness may deeply comprehend needs of their subordinates and achieve performance expectations through a high-quality exchange. High quality LMX between supervisors and subordinates may also well increase the responsiveness of the supervisors to the feedback from their subordinates and react with reasonable enhancement objectives and proper alterations in their attitudes and behaviors (Atwater et al., 1998; Atwater and Yammarino, 1997).

Leader-Member Exchange (LMX) theory provides a robust framework for understanding how high-quality relationships between leaders and followers can significantly enhance leadership self-awareness. In high-quality LMX relationships, leaders receive more frequent, honest, and constructive feedback from their followers, which serves as an important input for calibrating their self-perceptions (Dulebohn et al., 2012). The psychological safety inherent in these relationships creates an environment where followers feel more secure in providing straightforward opinions without fear of negative consequences, thus increasing the accuracy and benefit of feedback that leaders receive (Martin et al., 2016). Moreover, the trust-based nature of high-quality LMX relationships establishes a foundation where leaders are more receptive to potentially ego-threatening information, thereby facilitating greater alignment between self and other perceptions of leadership effectiveness (Lee and Carpenter, 2018).

Cognitive similarity leads to similar interpretation of information, shared understanding, effective communication, and mutually positive attitudes (Allinson et al., 2001; Armstrong et al., 2002, 2004). Mutual positive feelings increase the quality of exchange between leaders and their subordinates, and in turn, having a high LMX will help leaders understand how his/her leadership behaviors are perceived and interpreted by subordinates, in other words will increase their self-awareness. Leadership styles are shown to be reflections and combinations of leaders’ personalities and implicit leadership traits (Delbecq et al., 2013; Epitropaki et al., 2013; Foti et al., 2017; Lord and Dinh, 2014). Research also suggests that cognitive styles are important indicators of leadership styles (Hejazi, 2016). Recent meta-analyses conducted on leadership further propose that LMX is an important intervening mechanism for the leadership processes (Gottfredson and Aguinis, 2017).

The dyadic characteristic of LMX, which involves mutual influence between leaders and their subordinates, establishes an optimal conceptual framework for understanding how cognitive similarities promote reciprocal comprehension that enhances leader metacognition (Dulebohn et al., 2012). Unlike constructs such as trust or psychological safety, which are largely psychological in nature, LMX is a multifaceted relational structure that encompasses dimensions like affect, loyalty, contribution, and professional respect—factors that are directly impacted by cognitive alignment and, in turn, influence self-reflective leadership processes (Martin et al., 2016). Epitropaki et al. (2016) empirically validated that the social comparison dynamics present in LMX relationships create feedback mechanisms that significantly enhance leaders’ self-monitoring and behavioral adaptability, particularly when cognitive frameworks are aligned. Furthermore, the findings from the meta-analysis by Rockstuhl et al. (2012) indicate that LMX is strongly correlated with leadership outcomes across different cultures, highlighting its substantial explanatory strength in the dynamics of interpersonal leadership. Additionally, Matta and Van Dyne (2020) revealed that LMX effectively captures the bidirectional influence processes that enhance leader self-perception through interpersonal congruence, contrasting with alternative mediators that primarily illustrate unidirectional processes, which are inadequate for explaining the complex reciprocal dynamics involved in the evolution of leaders’ self-awareness.

The temporal dynamics of Leader-Member Exchange (LMX) further reinforce its significance as a mediating factor in the relationship between cognitive congruence and self-awareness, as it embodies an evolutionary process rather than a fixed entity. Recent longitudinal studies conducted by Cropanzano et al. (2017) revealed that LMX relationships evolve through specific phases that are particularly responsive to cognitive style alignment, thereby contributing to leaders’ ongoing development of self-insight. The resource exchange framework proposed by Lee et al. (2019) clarifies how LMX acts as a channel for information exchange between cognitively aligned pairs, enabling the flow of behavioral feedback that forms the foundation for leaders’ self-awareness. Notably, Erdogan and Bauer (2014) demonstrated that the quality of LMX serves as a moderator for the effectiveness of formal feedback mechanisms, illustrating how cognitive alignment fosters an environment conducive to productive feedback exchanges within high-quality LMX relationships. Finally, rigorous empirical work by Gottfredson et al. (2020) revealed that LMX uniquely encompasses both the affective and cognitive mechanisms through which interactional congruence influences self-perception accuracy.

On the basis of the aforementioned cases and rationales, it seems plausible to propose that cognitive style congruence between the supervisors and subordinates will increase LMX, and in turn LMX will increase supervisors’ self-awareness. Hence, this study also proposes:

*H2:* LMX between the supervisor and his/her subordinate will be positively related to supervisor’s self-awareness on his/her own leadership behaviors.

*H3:* LMX between the supervisor and his/her subordinate will mediate the relationship between the supervisor and his/her subordinate’s cognitive style congruence and the supervisor’s self-awareness.

## 3 Method

### 3.1 Samples and procedure

Data were gathered from white-collar employees and their supervisors employed in 36 organizations from various sectors in Turkey. We used structured questionnaires, which are comprised of measures already used and validated in the literature. Scales in the questionnaire were originally established in English and translated into Turkish using the back-translation technique (Brislin, 1980). Before applying the questionnaires, a pilot study was conducted to ensure that statements in the questionnaire were easily comprehended by white-collar employees.

To collect data, we employed a three-phase time-lagged design. We measured independent (i.e., cognitive styles of supervisors and subordinates) and control variables at Time 1 and dependent variables in the following phases. We captured mediator variable (i.e., leader-member exchange) at Time 2, which was 3 months after Time 1, and self-other agreement variables as outcomes (i.e., transformational leadership from supervisors as self- report and from their subordinates) at Time 3, which was 6 months after Time 2. Participants, especially subordinates, were kindly requested to desist from taking this survey in the following phases of the research, if their work arrangements and formal relationships with their supervisors had changed after the first survey.

Our research assistant applied questionnaires via face-to-face interviews on site for each time. Before administering the questionnaires, the purpose of the survey and the goal of the study were explained by our research assistant and added that participation was completely voluntary. Additionally, research assistant also explained and read the cover letter to ensure participants’ confidentiality. Participants were kindly requested to give the completed questionnaires back to the research assistant directly to assure their anonymity.

Of the 300 subordinates and 75 supervisors initially interviewed, at the end of third phase, we had usable questionnaires from 189 subordinates nested under 36 supervisors, generating a response rate of 63% for subordinates and 48% for supervisors. Whole set of analyzable data were available for 189 supervisor-subordinate dyads.

The average age of supervisors was 40 (s.d. of 7.3 years). The sample of supervisors was composed of 84% male and 95% of the supervisors were at least university graduates. Average tenure in years of the supervisors was 11 (s.d. of 8 years). The subordinates were 33 years old in average (s.d. of 7.4 years). The sample of subordinates was composed of 67% male and 86% of the subordinates were at least university graduates. Average tenure in years of the subordinates was 6.5 (s.d. of 5 years).

### 3.2 Measures

All statements in the scales were measured using a five-point Likert-type scale, where 1 indicates “strongly disagree” and 5 indicates “strongly agree.” Cronbach’s Alpha was used to assess reliability of scales. All the scales met the generally accepted reliability of 0.70 (Nunnally, 1978).

*Cognitive Styles:* To measure cognitive styles, we used 18-item Cognitive Style Indicator (CoSI), which was established and tested by Cools and Van den Broeck (2007). Cools and Van den Broeck (2007) modeled the three-dimensional version of cognitive styles scale and attained significant support for its construct validity. The CoSI differentiates three cognitive styles: knowing style (tapped by four items, e.g., “I like to analyze problems”), planning style (tapped by seven items, e.g., “I prefer clear structures to do my job”), and creating style (tapped by seven items, e.g., “I like to extend the boundaries”). Reliabilities of the cognitive style indicator are 0.78, 0.76 and 0.72 for the survey administered to subordinates and 0.83, 0.86 and 0.75 for the survey administered to supervisors, for knowing style, creating style and planning style, respectively.

*Leader-Member Exchange (LMX)*: LMX was assessed with LMX-7 scale adapted from the scales developed and tested by Graen and Uhl-Bien (1995). Sample items from the LMX scale include; “I would characterize my working relationship with my supervisor as exceptionally effective.” Validity and reliability of LMX-7 scale has been proven and wide usage of it in the leadership literature is shown and explained using several examples in recent studies (Gooty et al., 2012; Gottfredson and Aguinis, 2017; Matta et al., 2015). For this study, reliability of the LMX scale was 0.72.

*Leader Self-Awareness:* In this study, three transformational leadership dimensions, which were tapped with a 13-item scale established and tested by Podsakoff et al. (1990), were used to operationalize managerial self-awareness. In this scale, idealized influence was tapped by 5-items, individual support and intellectual stimulation were tapped by 4-items each. Since leader self-awareness questionnaire were asked to both supervisors and subordinates, statements were altered as required. Sample items include; “I have (My boss has) a clear understanding of where we are going” (idealized influence), “I behave (My boss behaves) in a manner thoughtful of my personal needs” (individual support) and “I challenge my subordinates (My boss challenges me) to think about old problems in new ways” (intellectual stimulation) (it shall be noted that “I” was replaced with “My boss” in the questionnaires administered to the subordinates). Reliabilities of the transformational leadership dimensions are 0.75, 0.85 and 0.78 for the survey administered to the subordinates and 0.66, 0.82 and 0.67 for the survey administered to the leaders, for idealized influence, individual support and intellectual stimulation, respectively.

### 3.3 Cross-level polynomial regressions and surface response analysis

We employed polynomial regression and surface response analysis to test our hypotheses (Edwards, 2002; Edwards et al., 2006). Those techniques were employed to more precisely investigate the exact characteristics of influence of supervisor-subordinate dyadic cognitive style congruence on dependent variables.

In our sample, the final data is comprised of multiple subordinates per supervisor which creates a multi-level structure. To confirm the nested structure of our data, we checked if the variability in outcome variables can be attributed to nesting of subordinates reporting to the same supervisor. The ICC(1) values (calculated as the ratio of between-group and total variance) were 0.18, χ^2^(35) = 41.51, *p* < 0.01 for LMX; 0.12, χ^2^(35) = 48.73, *p* < 0.01 for idealized influence; 0.11, χ^2^(35) = 39.86, *p* < 0.01 for intellectual stimulation; and 0.16, χ^2^(35) = 41.51, *p* < 0.01 for individual support; suggesting the use of multilevel analysis.

Thus, we utilized cross-level polynomial regressions as suggested by Jansen and Kristof-Brown (2005), which was also used in several research (e.g., Hu and Liden, 2013; Zhang et al., 2012).

In particular, the dependent variable (LMX) was regressed on five defined polynomial variables which are supervisors’ self-ratings on the cognitive style component (X), subordinates’ self-ratings on that same cognitive style component (Y), and second order terms as X^2^, X. Y, and Y^2^. To lessen multicollinearity, all predictor terms were scale-centered as proposed by Edwards (2002). A distinct polynomial regression was conducted for each of the three cognitive style components, which are knowing, planning and creating styles. As polynomial regression was employed, further analyses were performed to explore slopes and curvatures along the line of congruence (X = Y) and the line of incongruence (X = -Y).

However, since self-awareness as a dyadic congruence is included in the model as dependent variable, a dyadic congruence score was calculated for each supervisor- subordinate dyad utilizing the D-statistic. D-statistic is computed as the square root of the sum of the squared differences between supervisors’ ratings on their own leadership behaviors and subordinates’ ratings on their supervisors’ transformational leadership attitudes/behaviors. We multiplied the D-statistic score by −1 (minus one) so that a high score would indicate high level of similarity between the ratings of the supervisor and the subordinate. This approach allowed us to have self-awareness as a dependent variable in the proposed model.

### 3.4 Mediation test using the block variable approach

In order to explore the indirect influences of cognitive style (in)congruence on the outcome variables (i.e., self-awareness) through LMX (Hypothesis 3), we used the block variable approach suggested by Edwards and Cable (2009). Particularly, in order to acquire a single coefficient indicating the combined influence (i.e., congruence and incongruence influence) of the five polynomial terms (X, Y, X^2^, XxY, and Y^2^), we merged the five terms to generate a block variable which was a weighted linear composite. Once we built the block variable, we repeated the cross-level polynomial regression and calculated standardized regression coefficient for the block variable for mediation analysis. It is essential to emphasize that utilizing the block variable does not alter the calculated coefficients of other variables in the equation, nor the total explained variance (Edwards and Cable, 2009). We explored the statistical significance of the indirect effects by using bootstrapping (Efron and Tibshirani, 1993). We calculated bias-corrected confidence intervals for the indirect effects by bootstrapping 20,000 samples.

### 3.5 Results

Table 1 presents the means, standard deviations, intercorrelations, and reliability coefficients of the variables. Cognitive style components are moderately correlated with either each other or LMX and the outcome variables. Similarly, LMX is moderately correlated with difference scores calculated on supervisor and subordinate rated transformational leadership components.

**Table 1:** Descriptive statistics, reliabilities and intercorrelations among constructs.

	MEAN	ST. DEV.	SP COK	SP COC	SP COP	SB COK	SB COC	SB COP	LMX	IIN-D	IST-D	ISP-D
SP-COK	3.17	0.20	0.83a									
SP-COC	4.12	0.19	0.18*	0.86a								
SP-COP	4.09	0.22	0.65**	0.54**	0.75a							
SB-COK	4.20	0.15	−0.09	−0.11	−0.06	0.78a						
SB-COC	4.00	0.28	−0.05	−0.06	0.05	0.61**	0.76a					
SB-COP	4.13	0.27	−0.03	0.03	0.03	0.77**	0.59**	0.72a				
LMX	3.89	0.17	0.11*	0.07	0.09*	0.14*	0.10	0.12*	0.91a			
IIN-D	-	-	0.21**	0.07	0.06	−0.03	−0.01	−0.08	0.14*	1		
IST-D	-	-	0.02	0.03	0.10	0.04	−0.01	−0.06	0.16*	0.34**	1	
ISP-D	-	-	−0.04	0.15*	0.01	0.02	0.02	0.03	0.18*	0.28**	0.18*	1

**Parameter estimate is significant at the 0.01 level. *Parameter estimate is significant at the 0.05 level. a, Cronbach’s Alpha reliability; SP-COK, supervisor’s self-rated cognitive knowing style, SB-COK, subordinate’s self-rated cognitive knowing style,

SP-COC, supervisor’s self-rated cognitive creating style; SB-COC, subordinate’s self-rated cognitive creating style; SP-COP, supervisor’s self-rated cognitive planning style; SB-COP, subordinate’s self-rated cognitive planning style; LMX, subordinates’ perception on leader-member exchange.

IIN-D, D-statistic of dyadic supervisor-subordinate congruence on idealized infleunce.

IST-D, D-statistic of dyadic supervisor -subordinate congruence on intellectual stimulation; ISP-D, D-statistic of dyadic supervisor -subordinate congruence on individual support.

We performed CFA to assess the distinctiveness of the seven subordinate rated variables (i.e., knowing style, creating style, planning style, LMX, idealized influence, individual support and intellectual stimulation).

Before going further, we combined the items of the components into parcels for each distinct variable at a random order, as in earlier studies (e.g., Sass and Smith, 2006; Zhang et al., 2012). The hypothesized seven-factor model (Model 1 in Table 2) having unique yet interrelated (correlated) components was judged against various different models as alternatives. Table 2 depicts the model fit results. The proposed seven-factor model offers a sufficient fit [χ^2^ = 456.77, *p* < 0.01, df = 230, χ^2^/df = 1.98 (<3), CFI = 0.95; NFI = 0.90; TLI = 0.91; RMSEA = 0.03] and has considerably better fit compared with all other alternative models (Hair et al., 2006). Moreover, in the seven-factor model, all allots loaded significantly on their corresponding factors (having a *t*-value of 3.86 as lowest), supporting the convergent validity of scales. Discriminant validity is also attained for all factors as variance extracted for each factor is larger than their squared correlations with other factors (Fornell and Larcker, 1981). Given these CFA results, we carried on our analyses considering these variables as distinct factors.

**Table 2:** Model fit results for confirmatory factor analyses.

Model	Paths added to resulting model	χ^2^	df	χ^2^/df	CFI	NFI	TLI	RMSEA
Model 1	Hypothesized seven-factor model	456.77	230	1.98	0.95	0.90	0.91	0.03
Model 2	Five-factor model (Supervisors’ COK, COP and COC are combined)	893.42	241	3.71	0.84	0.80	0.78	0.08
Model 3	Five-factor model (Subordinates’ COK, COP and COC are combined)	905.55	241	3.75	0.72	0.73	0.64	0.10
Model 4	Three-factor model (Supervisors’ COK, COP and *C*OC are combined, as well as Subordinates’ COK, COP and COC are combined)	1022.83	249	4.11	0.69	0.68	0.62	0.12
Model 5	Single-factor model	1309.37	254	5.15	0.62	0.62	0.58	0.15

All χ^2^ are significant at *p* < 0.01. CFI, Comparative Fit Index; TLI, Tucker-Lewis Index; NFI: Normed Fit Index; RMSEA, Root-mean square error of approximation.

Our first hypothesis proposed that congruence between supervisors’ and subordinates’ cognitive styles will have a positive influence on subordinates’ perception on LMX. In Table 3, first column shows the predicted coefficients and significance values on congruence and incongruence lines for the cross-level polynomial regressions in predicting LMX. As shown in Table 3, polynomial regression analyses revealed that the three second-order polynomial terms were statistically significant for only two cognitive style components, which are cognitive knowledge style (*F* = 21.82, *p* < 0.01) and cognitive creating style (*F* = 19.22, *p* < 0.01).

**Table 3:** Results of cross-level polynomial regression analyses.

	Dependent variable LMX		Dependent variable LMX		Dependent variable LMX
Variables (Knowing style)	*β*	Variables (Planning style)	*β*	Variables (Creating style)	*β*
Constant	3.48	Constant	3.24	Constant	3.54
SP-COK (X)	0.26**	SP-COP (X)	0.14*	SP-COC (X)	0.28**
SR-COK (Y)	0.42**	SR-COP (Y)	0.34**	SR-COC (Y)	0.44**
X^2^	0.08*	X^2^	0.14	X^2^	0.18*
X x Y	0.06*	X x Y	0.08	X x Y	0.09*
Y^2^	−0.14*	Y^2^	0.07	Y^2^	−0.12*
F (Note)	21.82**	F (Note)	12.25**	F (Note)	19.22**
R^2^	0.43	R^2^	0.24	R^2^	0.24
a1 Slope along X = Y	0.68**	a1 Slope along X = Y	0.11	a1 Slope along X = Y	0.72**
a2 Curvature along X = Y	0.02	a2 Curvature along X = Y	0.02	a2 Curvature along X = Y	0.15
a3 Slope along X = -Y	−0.26*	a3 Slope along X = -Y	−0.14	a3 Slope along X = -Y	−0.16*
a4 Curvature along X = -Y	−0.12	a4 Curvature along X = -Y	−0.05	a4 Curvature along X = -Y	−0.03
Within-group variance (σ^2^)	0.39	Within-group variance (σ^2^)	0.42	Within-group variance (σ^2^)	0.40
Between-group variance (τ^2^)	0.03	Between-group variance (τ^2^)	0.08	Between-group variance (τ^2^)	0.04
R1^2^	0.35	R1^2^	0.23	R1^2^	0.33

**Parameter estimate is significant at the 0.01 level. *Parameter estimate is significant at the 0.05 level.

SP-COK, supervisor’s self-rated cognitive knowing style; SR-COK, subordinate’s self-rated cognitive knowing style; SP-COC, supervisor’s self-rated cognitive creating style; SR-COC, subordinate’s self-rated cognitive creating style; SP-COP, supervisor’s self-rated cognitive planning style; SR-COP, subordinate’s self-rated cognitive planning style; LMX, subordinates’ perception on leader-member exchange.

R1^2^ is calculated as the variance accounted for in LMX (Yij) by level-1 predictors (cognitive styles) using the measure proposed by Snijders and Bosker (2012).

**Figure 2:**
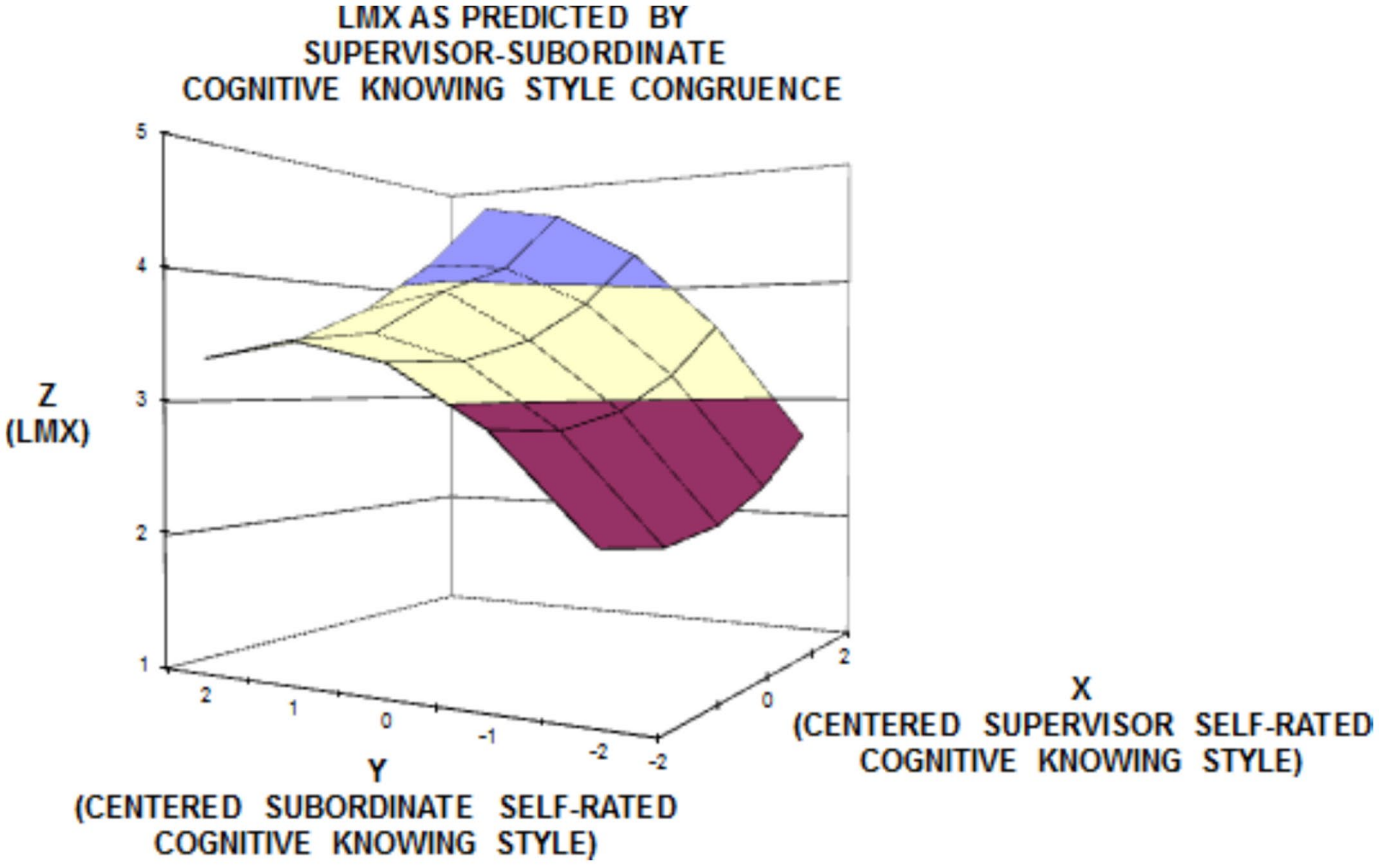
Surface response analysis for the influence of congruence between supervisor’s self-rated and subordinate’s self-rated cognitive knowing style on subordinate self-rated LMX.

**Figure 3:**
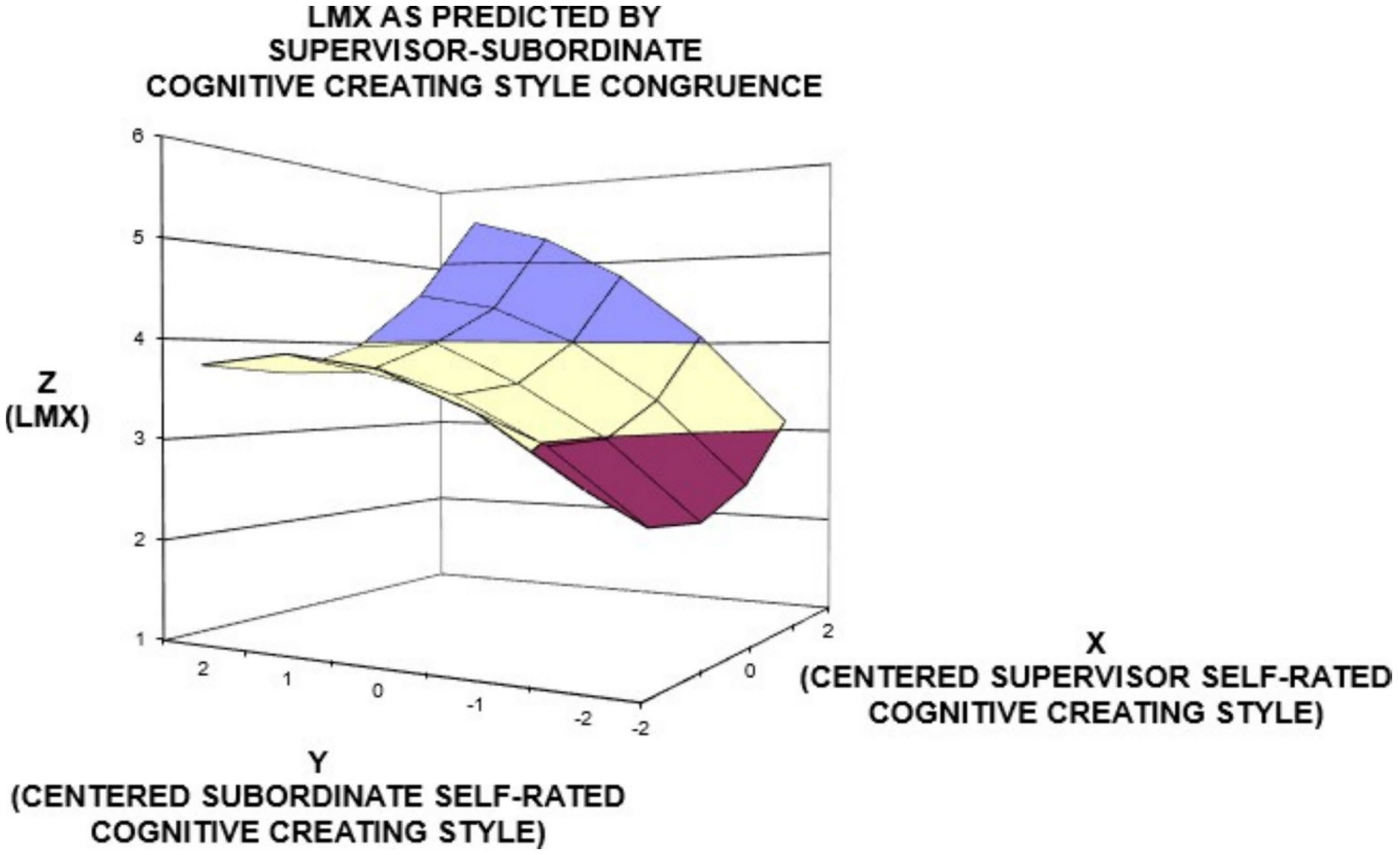
Surface response analysis for the influence of congruence between supervisor’s self-rated and subordinate’s self-rated cognitive creating style on subordinate self-rated LMX.

In order to better understand the varying influences of (in)congruence of supervisor’s and subordinate’s cognitive styles, we also performed surface response analyses. Three- dimensional (3-D) diagrams of the regarding surface response analyses are presented in Figures 2, 3.

By assessing the surface plots of knowing style (Figure 2) and creating style (Figure 3), we found that LMX was at the highest level when both supervisors’ and subordinates’ ratings are high (in agreement/good) and at the second highest level when subordinates’ ratings are higher than supervisors’ ratings (underestimator supervisor) (shown by the left corners of graphics). Contrarily, LMX was at a lower level when subordinates’ ratings are lower than supervisors’ ratings (overestimator supervisor) and at the lowest level when the components were rated as low both by the supervisor and the subordinate (in agreement/poor) (shown by the right corners of graphics). Since second-order polynomial terms for the congruence of planning style yielded that the regression coefficients of the relevant equation were not statistically significant (Table 3), its graphical illustrations are not depicted.

**Figure 4:**
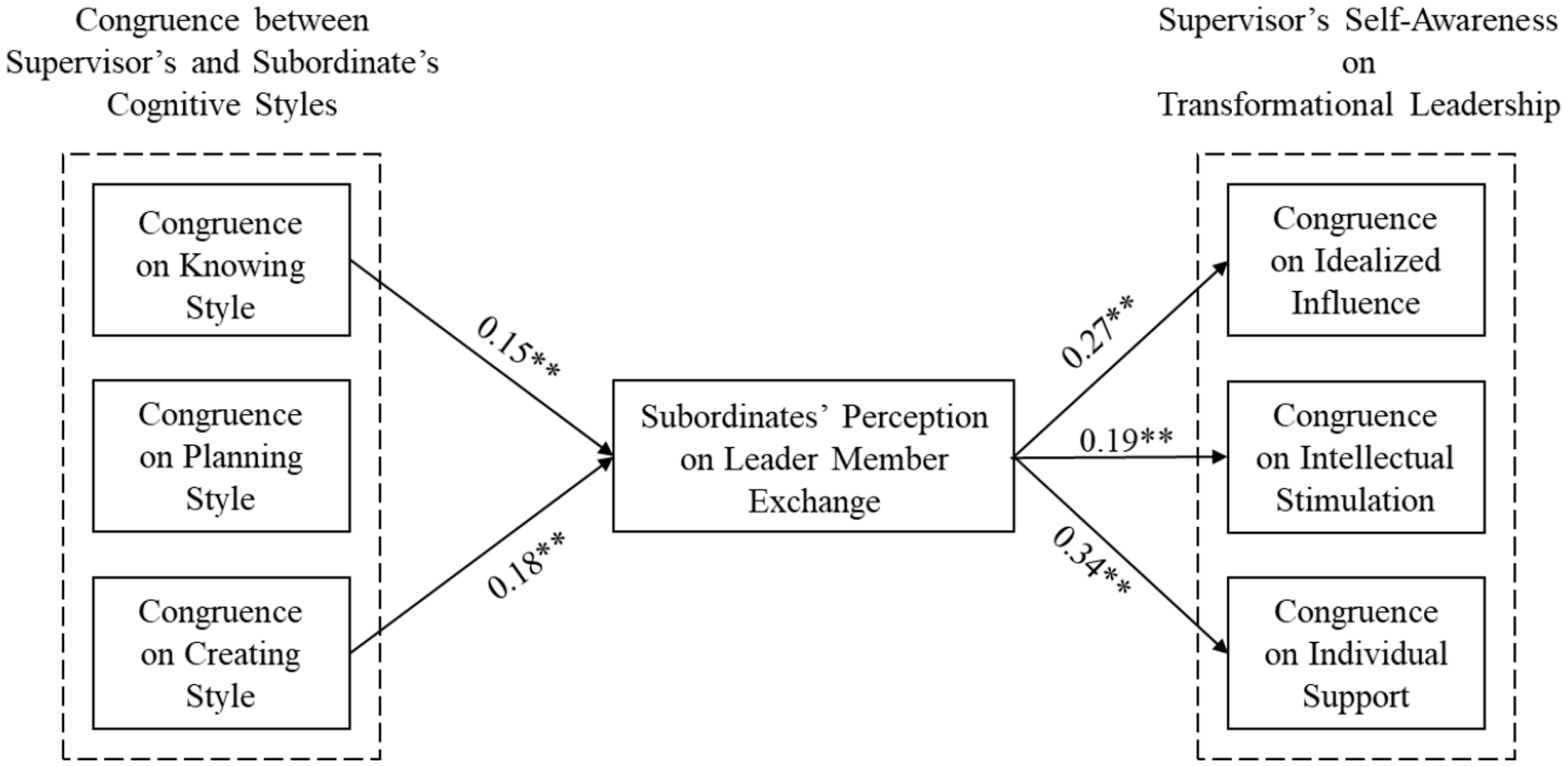
Resulting research model (Only significant relationships are depicted on the figure).

To assess the mediating role of LMX, we administered the block variable approach to attain a single coefficient indicating the joint effect of supervisor-subordinate rated knowing style and creating style on LMX. The standardized coefficient of the joint influence on LMX is 0.15 (*p* < 0.01; Table 4) for cognitive knowing style and 0.18 (*p* < 0.01; see Table 4) for cognitive creating style. Path coefficient on LMX for cognitive planning style is insignificant (0.09, *p* > 0.05). Table 4 presents the results of the mediation analyses. Figure 4 presents the final significant model.

**Table 4:** Results from tests of direct and indirect effects of congruence/incongruence in cognitive styles on supervisor’s self-awareness.

Variables	LMX	IIN-D	IST-D	ISP-D
Coefficient of the block variable (i.e., direct effect of congruence on cognitive knowing style) (CBV_COK)	0.15**	0.11	0.07	0.09
Coefficient of the block variable (i.e., direct effect of congruence on cognitive planning style) (CBV_COP)	0.09	0.03	0.02	0.05
Coefficient of the block variable (i.e. direct effect of congruence on cognitive creating style) (CBV_COC)	0.18**	0.08	0.06	0.09
Coefficient of LMX (γLMX)	-	0.27**	0.19**	0.34**
Indirect effect of congruence on cognitive knowing style via LMX (= CBV_COK x γLMX) [95% bootstrapped confidence intervals for the indirect effect]	-	0.04* [0.01, 0.07]	0.03* [0.01, 0.010]	0.05* [0.03, 0.011]
Indirect effect of congruence on cognitive planning style via LMX (= CBV_COP x γLMX) [95% bootstrapped confidence intervals for the indirect effect]	-	0.02 [0.01, 0.04]	0.02 [0.01, 0.06]	0.03 [0.01, 0.07]
Indirect effect of congruence on cognitive creating style via LMX (= CBV_COC x γLMX) [95% bootstrapped confidence intervals for the indirect effect]	-	0.05* [0.02, 0.09]	0.03* [0.01, 0.09]	0.06* [0.03, 0.12]

Standardized coefficient are reported.

**Parameter estimate is significant at the 0.01 level. *Parameter estimate is significant at the 0.05 level.

As presented in Table 4, the direct influences of (in)congruence in cognitive styles on the resulting variables are all insignificant when LMX is in the equation. The indirect effects of cognitive style (in)congruence on self-other agreement, via LMX, are significant from knowing style to idealized influence [0.04, *p* < 0.05, 95% CI = (0.01, 0.07)], to individual stimulation [0.03, *p* < 0.05, 95% CI = (0.01, 0.10)], and to individual support [0.05, *p* < 0.01, 95% CI = (0.03, 0.11)]; are also significant from creating style to idealized influence [0.05, *p* < 0.05, 95% CI = (0.02, 0.09)], to individual stimulation [0.03, *p* < 0.05, 95% CI = (0.01, 0.09)], and to individual support [0.06, *p* < 0.05, 95% CI = (0.03, 0.12)]. Yet, indirect effects of planning style are insignificant for all three self-other agreement components; i.e. on idealized influence [0.02, *p* > 0.05, 95% CI = (0.01, 0.04)], on individual stimulation [0.02, *p* > 0.05, 95% CI = (0.01, 0.06)], and on individual support [0.03, *p* > 0.05, 95% CI = (0.01, 0.07)]. Considering the direct and indirect influences, findings reveal that LMX fully mediated the joint effects of supervisor-subordinate cognitive style congruence on supervisor rated self-other agreement on transformational leadership (partially supports Hypothesis 3 suggesting that LMX fully mediates the association between cognitive style (in)congruence and the self-other agreement).

### 3.6 Controlling for endogeneity

In order to test our model, we collected data at three different time points and from two different sources. Nevertheless, nonexperimental designs cannot completely rule out the possible threat of endogeneity and ensure that the causality links are fully established.

In our study, given that LMX is not an exogenous variable in nature and thus does not vary independently of such other potential causes, corrective statistical procedures should be undertaken to assure that the endogeneity does not pose an important threat (Antonakis, 2017; Antonakis et al., 2010, 2014). As suggested by Cools et al. (2014), controlling for possible omitted predictors is needed before the cognitive style constructs can be assuredly used in practice. In order to deal with those endogeneity concerns, we analyzed our data using two-stage least squares with instrumental variables (IV) (Antonakis et al., 2014). Individual differences might present a possible solution provided that they are mostly exogenous, and thus can be useful in congruence models of leadership (Antonakis et al., 2012). Also, research has clearly noted that cognitive style measures must show incremental variance beyond personality (Von Wittich and Antonakis, 2011). Thus, we decided to instrument supervisors’ and subordinates’ personality (IV). To measure personality (at Time 1), we used the 44-item Big Five Inventory (John and Srivastava, 1999) to measure the five broad personality traits. All endogenous variables, specifically cognitive style constructs and LMX, were regressed on exogenous variable personality.

The two-stage least-square analysis confirmed our predictions; coefficients of big-five personality traits were not significant (For supervisors’ personality: *β* = 0.02 for neuroticism, ns; *β* = 0.08 for extraversion, ns; *β* = 0.07 for openness, ns; *β* = 0.06 for agreeableness, ns; *β* = 0.12 for conscientiousness, ns; For subordinates’ personality: *β* = 0.00 for neuroticism, ns; *β* = 0.01 for extraversion, ns; *β* = 0.02 for openness, ns; *β* = 0.01 for agreeableness, ns; *β* = 0.03 for conscientiousness, ns). Those results were reconfirmed when we run the Durbin–Wu– Hausman test, which failed to reject the null hypothesis for relationships between congruence on knowing style and LMX (0.17; ns), congruence on planning style and LMX (0.86; ns), and congruence on creating style and LMX (0.62; ns). The test also failed to reject the null hypotheses for the LMX-self-other agreement on idealized influence (0.43; ns), LMX-self- other agreement on intellectual stimulation (0.22; ns), and LMX-self-other agreement on individual support (1.12; ns) relationships. Moreover, an IV model may yield biased results if weak instruments are used (Antonakis et al., 2014). Hence, overall these results suggest that there is no clear risk of endogeneity in our study.

## 4 Discussion and conclusion

By this research, we explored the relationship among supervisor/subordinate cognitive style congruence, subordinate rated leader-member exchange and supervisor’s managerial self-awareness, in other words self-other agreement, which was measured as the congruence between the supervisors’ self-rating on their own transformational leadership behaviors and subordinates’ ratings on their supervisors’ transformational leadership behaviors. We hypothesized that the cognitive congruence between the supervisor and the subordinate would positively be related to supervisor’s managerial self-awareness, and LMX would mediate that relationship. Cognitive congruence and managerial self-awareness in our study were measured by dyadic data collected from supervisors and their subordinates.

Findings yield that congruence in two cognitive styles, namely knowing style and creating style are positively related to LMX, no significant link was found between congruence on planning style and LMX. People with knowing style rely on the facts, figures and focus on logical thinking. People with creating style are divergent thinkers and usually seek for new and different ideas and approaches. Knowing and creating styles together stimulate thinking about problems in innovative ways, challenging employees with original and novel ideas and pushing them to find new and creative solutions. Thus, when both supervisor and subordinate have knowing and/or creating cognitive styles, supervisor would show attitudes and behaviors that stimulate information sharing and innovation and the subordinates perceive those behaviors as motivating. Because people having knowing and/or creating styles also prefer open communication, they would have a high-quality relationship between each other.

Contrary to expectations, the link between congruence on cognitive planning style and LMX is found to be statistically insignificant. People high in planning style, also called planners, like structured approaches and usually are goal oriented rather than being people oriented. LMX, on the other hand, is related to the emotional aspect of leadership and also deals with having respect and showing consideration to other people’s feelings. Since planners usually focus on goals rather than people, they would not show any concern regarding interpersonal relationships and most probably would not care the quality of their relationship. Research has also revealed that individuals high in planning style tend to favor structured, goal-oriented approaches over relationally focused behavior (Ameden et al., 2024). This cognitive-behavioral orientation is closely associated with the personality trait of planfulness, a recent behavioral construct grounded in conscientiousness but more precisely reflecting one’s tendency to think ahead, strategize, and sequence actions to achieve long-term objectives (Ludwig et al., 2018). Empirical findings indicate that planfulness significantly predicts goal achievement, emphasizing its role in task-focused behavior (Ludwig et al., 2019). Furthermore, planners are more likely to employ proactive self-regulatory strategies, such as preemptive coping, which prioritize efficiency and task completion over interpersonal engagement (Ameden et al., 2024). This orientation often results in a weakened emphasis on social interaction, as planners channel cognitive resources toward optimizing goal-directed actions rather than enhancing relational dynamics.

Findings of this research reveal that LMX has a significant role in enhancing self- awareness of supervisors regarding their leadership behaviors. This result implies the importance of LMX and gives a strong indication why fostering LMX should be addressed in leadership development programs (also see Martin et al., 2016).

Findings indicate that; when supervisors have good relationships and high-quality social and informational exchange with their subordinates, they will be aware of how their own leadership is perceived and interpreted by their subordinates. Through their relationship and exchange, supervisors can influence their subordinates, communicate their vision, show their support and care and lead their subordinates toward the right direction for the organization. By having an information flow, they will have better competency to interpret the reactions of their subordinates. Through this high-quality relationship with their subordinates, supervisors will also promote their subordinates’ positive perceptions regarding leadership performance and foster positive work-related attitudes and behaviors, such as higher organizational commitment and lower turnover intentions.

Epitropaki and Martin (2005) demonstrate that LMX is positively related to the high degree of fit between actual supervisor attributes and followers’ perceptions on their leadership skills. Furthermore, when both supervisors and subordinates possess common characteristics and perceptions, favorable perceptions is most likely to develop and contribute to constructive work-related outcomes, such as higher performance, higher job satisfaction and low turnover (intentions) (Epitropaki and Martin, 2005). It is crucial to promote self- awareness of supervisors, among other leadership competencies. Hence, supervisors are strongly recommended to thoroughly focus on inconsistencies and differentiations between their self-assessments and others’ assessments on their leadership behaviors. Studies have provided strong support on the fact that supervisors tend to alter their attitudes and behaviors even substantially, when they get worse assessments from others compared to their own self- assessments (e.g., Johnson and Ferstl, 1999).

To foster self-other agreement, it is also very important to explore the possible reasons of inconsistencies between supervisor and subordinates’ assessments. Feedback- seeking behaviors might be considered a suitable management strategy to enhance self- awareness (Devloo et al., 2011). Supervisors who deliberately and proactively seek feedback from their own superiors, subordinates and peers, can acquire significant information regarding their own leadership performance and use that information to adjust their attitudes and behaviors in a better way to enhance their future performance.

### 4.1 Theoretical implications

From a theoretical perspective, Social Identity Theory (SIT) elucidates how the congruence of cognitive styles between supervisors and their subordinates can impact the self-awareness of supervisors by shaping their social identity in the organizational setting. The empirical findings regarding cognitive style congruence and supervisors’ self-awareness can be interpreted through the social identity mechanisms articulated in Social Identity Theory (SIT). According to SIT, individuals derive a considerable portion of their self-concept from the groups they are part of, including their work groups or organizational units (Albert et al., 2000; Hogg and Terry, 2000). The theory posits that individuals endeavor to maintain a favorable social identity by comparing their in-group positively against out-groups. When there is cognitive style congruence between supervisors and subordinates, they are more likely to identify as members of the same social identity within the organization. SIT indicates that an individual’s self-concept and self-awareness are influenced by their group affiliations and social identity. In the context of supervisor-subordinate relationships, a strong social identity, facilitated by cognitive style congruence, could enhance the supervisor’s self-awareness through improved interpersonal relationships and rapport.

Consistent with SIT’s premise that individuals derive aspects of their self-concept from membership in social groups (Hogg and Terry, 2000), congruence in knowing and creating cognitive styles likely facilitates psychological processes of social categorization, whereby supervisors and subordinates perceive one another as sharing a cognitive in-group identity. This shared categorization would have an important effect on information exchange processes that are vital to leaders’ self-awareness development. As demonstrated by Steffens et al. (2014), when leaders and followers identify themselves as members of the same group, the frequency and quality of communication increases substantially, which provides leaders with more accurate behavioral feedback as an important input for developing their self-awareness. The findings also align with Epitropaki et al. (2017), who established that perceived cognitive similarity between leaders and followers strengthens implicit leadership theories congruence, thereby enhancing the quality of the categorization processes that underpin leader identity internalization and subsequent self-awareness. Furthermore, the specific significance of knowing and creating styles can be contextualized within Guillén et al.'s (2015) findings that identity-based leadership is most effective when the shared identity attributes align with organizationally valued competencies, such as knowledge acquisition and creative innovation in contemporary organizational contexts.

The full mediation effect of LMX in the relationship between cognitive style congruence and leaders’ self-awareness can be understood through the social identity mechanism of interpersonal attraction, whereby shared group membership engenders positive affect and enhanced relationship quality. Cognitive similarity serves as a potent antecedent for social attraction, which manifests in organizational contexts as enhanced LMX quality. Within these high-quality exchange relationships, subordinates are more likely to engage in identity-affirming behaviors toward supervisors with congruent cognitive styles, including providing constructive feedback that enhances leader self-awareness (Sluss et al., 2012). The significant mediating role of LMX also supports the findings of Steffens et al. (2021), who established that shared social identity facilitates leadership influence through relational mechanisms that transform cognitive similarity into enhanced mutual understanding, thereby providing leaders with the psychological resources necessary to develop accurate self-awareness regarding their transformational leadership behaviors.

On the other hand, transformational leadership is still considered as the most effective leadership style in today’s complex and dynamic business environment (Gottfredson and Aguinis, 2017). Hence, self-other agreement happens to be more vital, since Transformational leadership also encompasses intense interpersonal communication between supervisors and their subordinates. When there are misunderstandings and discrepancies arising from insufficient communication, this makes it so difficult for the supervisor to develop and sustain an effective leadership. As a consequence, having a high level of self-awareness is very crucial for the supervisors to promote and effectively use their transformational leadership skills.

### 4.2 Managerial implications

The results of this study present several important implications for organizational practices and the development of leadership. Firstly, it is recommended that organizations utilize cognitive style assessment tools during the recruitment and team-building phases to strategically align supervisors and subordinates with compatible cognitive styles. This alignment has been shown to notably improve the quality of leader-member exchanges and enhance leaders’ self-awareness regarding their transformational leadership behaviors (Zhang and Sternberg, 2005). Secondly, leadership development initiatives should be restructured to include training on the appreciation of cognitive diversity and adaptive communication techniques. These modules would equip leaders to engage effectively with subordinates who possess diverse cognitive styles, thereby promoting high-quality leader-member exchange relationships even when complete cognitive style alignment is not feasible. Thirdly, organizations ought to implement structured feedback systems that focus on cognitive style dimensions, allowing leaders to cultivate metacognitive awareness of how their information processing preferences influence their leadership effectiveness and the quality of their relationships with subordinates. Lastly, the mediating role of leader-member exchange in the connection between cognitive style congruence and leaders’ self-awareness indicates that organizations should emphasize relationship-building strategies as a practical means to improve leadership effectiveness, rather than solely concentrating on individual leader characteristics. This approach is supported by meta-analytic findings that highlight the essential role of high-quality exchange relationships in achieving positive organizational outcomes (Dulebohn et al., 2012).

## 5 Strengths, limitations and future recommendations

One of the most important strengths of this study is its time-lagged design in collecting dyadic data from multiple sources (i.e., supervisors and subordinates). We gathered LMX measures 3 months after measuring the independent variables (i.e., cognitive styles of supervisors and subordinates), and gathered leadership measures 6 months after that. This time-lagged design allowed us to lessen serious concerns about common source and common method bias as potential threats for our study (Podsakoff et al., 2003). Another strength of this study comes from using multilevel analysis, which allowed us to appropriately account for the nested nature of the data. Moreover, we employed a multilevel polynomial regression and surface response analysis (e.g., Edwards, 2002), to test the effects of (in)congruence between cognitive styles of subordinates and supervisors.

In our study, we used self-report measures that have several advantages in capturing subjective perceptions of leadership. These instruments allow respondents to convey their personal evaluations, emotional responses, and cognitive interpretations of leader behavior, which are critical in understanding constructs such as transformational leadership and leader-member exchange (LMX), especially in self-awareness concept. Despite concerns regarding common method bias and social desirability, when employed with appropriate methodological safeguards, such as time-lagged design, dyadic data from multiple sources (from supervisors and subordinates) in a multi-level setting, anonymity, as well as other statistical controls, self-reports remain a valuable and valid tool for explaining the psychological mechanisms underlying leadership perceptions.

However, this study should be considered with some methodological boundaries in mind. First, supervisors and subordinates constituting our sample was chosen from the organizations in Turkey. So, we suggest the replication of similar studies in diverse cultural settings, which would enhance the robustness and applicability of findings, as well as extend the external validity of the results. Especially cross-cultural explorations of the relationships in our research model might be helpful to clarify how the associations among cognitive styles, social exchanges and self-other agreement forms in different context.

Second, the design of this study could not exclude the effects of common-method bias for the subordinates’ perceptions, since data collected from subordinates for LMX and the leadership of their supervisors as self-report (Podsakoff et al., 2003). However, the results of the CFA common latent factor test suggested that common method variance was not of great concern and thus was unlikely to confound the interpretations of results (Richardson et al., 2009).

This study has focused on supervisors’ self-awareness on their transformational leadership. Thus, future studies may extend this research by considering other leadership approaches, e.g., servant leadership, authentic leadership, etc. Besides, future studies may also focus on moderating and mediating influence of different attitudinal and behavioral components, such as trust, communication, teamwork, ethical climate, value congruence, person-organization fit, etc. To eliminate methodological considerations, future research may also be conducted using longitudinal data and observe how self-awareness changes over time along with the change in antecedent, mediator or moderator variables.

We also recommend for future studies including different moderating variables, such as tenure, organizational culture, leader experience or alternative constructs into the model. Having moderators or significant control variables in the model could broaden the scope of the study and also could provide alternative explanations of the relationship between the variables from the cultural or industrial perspective.

Even with these limitations, it is believed that the study achieved its primary purpose and substantially contributed to the literature. Our results indicate that statistically significant associations among cognitive congruence between the supervisor and his/her subordinate, LMX and the supervisor’s managerial self-awareness. Thus, some level of congruence between supervisors’ and their subordinates’ cognitive styles is needed to create a high- quality exchange. Furthermore, in order to be able to foster leaders’ self-awareness, there should be a good level of social and informational exchange between supervisors and their subordinates, in addition to their congruence on cognitive styles.

## Data Availability

The datasets used in this study are available from the corresponding author upon request.

## References

[ref1] Akdere M. Top M. Tarcan M. (2021). The impact of transformational leadership on employee attitudes: implications for human resources. MEJM 8, 611–629. doi: 10.1504/MEJM.2021.118456

[ref2] Albert S. Ashforth B. E. Dutton J. E. (2000). Organizational identity and identification: charting new waters and building new bridges. Acad. Manag. Rev. 25, 13–17. doi: 10.5465/amr.2000.2791600

[ref3] Al-Kenane K. Almoraish A. Al-Enezi D. Al-Matrouk A. AlBuloushi N. AlReshaid F. (2025). The process through which young adults form attitudes towards sustainable products through social media exposure in Kuwait. Sustain. For. 17:4442. doi: 10.3390/su17104442

[ref4] Alkharafi N. Alsabah M. (2025). Globalization: an overview of its main characteristics and types, and an exploration of its impacts on individuals, firms, and nations. Economies 13:91. doi: 10.3390/economies13040091

[ref5] Allinson C. W. Armstrong S. J. Hayes J. (2001). The effects of cognitive style on leader- member exchange: a study of manager-subordinate dyads. J. Occup. Organ. Psychol. 74, 201–219. doi: 10.1348/096317901167316

[ref6] Ameden W. C. Tricomi E. Heintzelman S. J. (2024). The role of planfulness for well-being, stress, and goal disruption during COVID-19. Front. Psychol. 15:1224451. doi: 10.3389/fpsyg.2024.1224451, PMID: 38390411 PMC10881737

[ref7] Antonakis J. (2017). On doing better science: from thrill of discovery to policy implications. Leadersh. Q. 28, 5–21. doi: 10.1016/j.leaqua.2017.01.006

[ref8] Antonakis J. Bendahan S. Jacquart P. Lalive R. (2010). On making causal claims: a review and recommendations. Leadersh. Q. 21, 1086–1120. doi: 10.1016/j.leaqua.2010.10.010, PMID: 40433219

[ref9] Antonakis J. Bendahan S. Jacquart P. Lalive R. (2014). “Causality and endogeneity: problems and solutions” in The Oxford handbook of leadership and organizations. ed. Day D. V. (New York: Oxford University Press), 93–117.

[ref10] Antonakis J. Day D. V. Schyns B. (2012). Leadership and individual differences: at the cusp of a renaissance. Leadersh. Q. 23, 643–650. doi: 10.1016/j.leaqua.2012.05.002

[ref11] Armstrong S. J. Allinson C. W. Hayes J. (2002). Formal mentoring systems: an examination of the effects of mentor/protege cognitive styles on the mentoring process. J. Manage. Stud. 39, 1111–1137. doi: 10.1111/1467-6486.00326

[ref12] Armstrong S. J. Allinson C. W. Hayes J. (2004). The effects of cognitive style on research supervision: a study of student-supervisor dyads in management education. Acad. Manage. Learn. Educ. 3, 41–63. doi: 10.5465/AMLE.2004.12436818

[ref13] Armstrong S. J. Sadler-Smith E. (2006). “Cognitive style and its relevance for the management of careers” in Paper presented at the 66th conference of the academy of management (August, Atlanta, GA).

[ref14] Ashford S. J. Cummings L. L. (1983). Feedback as an individual resource: personal strategies of creating information. Organ. Behav. Hum. Perform. 32, 370–398. doi: 10.1016/0030-5073(83)90156-3

[ref15] Ashforth B. E. Mael F. (1989). Social identity theory and the organization. Acad. Manag. Rev. 14, 20–39. doi: 10.2307/258189

[ref16] Atwater L. Ostroff C. Yammarino F. Fleenor J. (1998). Self‐other agreement: does it really matter? Pers. Psychol. 51, 577–598. doi: 10.1111/j.1744-6570.1998.tb00252.x

[ref17] Atwater L. Waldman D. Ostroff C. Robie C. Johnson K. M. (2005). Self-other agreement: comparing its relationship with performance in the U.S. and Europe. Int. J. Sel. Assess. 13, 25–40. doi: 10.1111/j.0965-075X.2005.00297.x

[ref18] Atwater L. Wang M. Smither J. W. Fleenor J. W. (2009). Are cultural characteristics associated with the relationship between self and others’ ratings of leadership? J. Appl. Psychol. 94, 876–886. doi: 10.1037/a0014561, PMID: 19594231

[ref19] Atwater L. E. Yammarino F. J. (1992). Does self‐other agreement on leadership perceptions moderate the validity of leadership and performance predictions? Pers. Psychol. 45, 141–164. doi: 10.1111/j.1744-6570.1992.tb00848.x

[ref20] Atwater L. Yammarino F. (1997). Self-other agreement: a review and model. Res. Personnel Hum. Resour. Manage. 15, 121–174.

[ref21] Avolio B. J. (2007). Promoting more integrative strategies for leadership theory building. Am. J. Psychol. 62, 25–33. doi: 10.1037/0003-066X.62.1.25, PMID: 17209677

[ref22] Avolio B. J. Walumbwa F. O. Weber T. J. (2009). Leadership: current theories, research, and future directions. Annu. Rev. Psychol. 60, 421–449. doi: 10.1146/annurev.psych.60.110707.163621, PMID: 18651820

[ref23] Avolio B. J. Zhu W. Koh W. Bhatia P. (2004). Transformational leadership and organizational commitment: mediating role of psychological empowerment and moderating role of structural distance. J. Organ. Behav. 25, 951–968. doi: 10.1002/job.283

[ref24] Aycan Z. Kanungo R. N. Mendonca M. Yu K. Deller J. Stahl G.. (2000). Impact of culture on human resource management practices: a 10‐country comparison. Appl. Psychol. 49, 192–220. doi: 10.1111/1464-0597.00010

[ref25] Banks G. C. McCauley K. D. Gardner W. L. Guler C. E. (2016). A meta-analytic review of authentic and transformational leadership: a test for redundancy. Leadersh. Q. 27, 634–652. doi: 10.1016/j.leaqua.2016.02.006

[ref26] Berson Y. Sosik J. J. (2007). The relationship between self-other rating agreement and influence tactics and organizational processes. Group Organ. Manag. 32, 675–698. doi: 10.1177/1059601106288068

[ref27] Brigham K. H. de Castro J. O. Shepherd D. A. (2007). A person–organization fit model of owner-managers’ cognitive style and organizational demands. Entrep. Theory Pract. 31, 29–51. doi: 10.1111/j.1540-6520.2007.00162.x

[ref28] Brislin R. W. (1980). “Translation and content analysis of oral and written materials” in Handbook of cross-cultural psychology. eds. Triandis H. C. Berry J. W., vol. 2 (Boston: Allyn & Bacon), 389–444.

[ref29] Caldwell C. Hayes L. A. (2016). Self-efficacy and self-awareness: moral insights to increased leader effectiveness. J. Manage. Dev. 35, 1163–1173. doi: 10.1108/JMD-01-2016-0011

[ref30] Castro C. B. Perinan M. M. V. Bueno J. C. C. (2008). Transformational leadership and followers’ attitudes: the mediating role of psychological empowerment. Int. J. Hum. Resour. Manag. 19, 1842–1863. doi: 10.1080/09585190802324601

[ref31] Chilton M. A. Hardgrave B. C. Armstrong D. J. (2005). Person–job cognitive style fit for software developers: the effect on strain and performance. J. Manag. Inf. Syst. 22, 193–226. doi: 10.1080/07421222.2005.11045849

[ref32] Church A. (1997). Managerial self-awareness in high-performing individuals in organizations. J. Appl. Psychol. 82, 281–292. doi: 10.1037/0021-9010.82.2.281, PMID: 9109286

[ref33] Cools E. Armstrong S. J. Verbrigghe J. (2014). Methodological practices in cognitive style research: insights and recommendations from the field of business and psychology. Eur. J. Work Organ. Psychol. 23, 627–641. doi: 10.1080/1359432X.2013.788245

[ref34] Cools E. Van den Broeck H. (2007). Development and validation of the cognitive style indicator. J. Psychol. 141, 359–387. doi: 10.3200/JRLP.141.4.359-388 17725071

[ref35] Cools E. Van den Broeck H. Bouckenooghe D. (2009). Cognitive styles and person- environment fit: investigating the consequences of cognitive (mis)fit. Eur. J. Work Organ. Psychol. 18, 167–198. doi: 10.1080/13594320802295540

[ref36] Cropanzano R. Dasborough M. T. Weiss H. M. (2017). Affective events and the development of leader-member exchange. Acad. Manag. Rev. 42, 233–258. doi: 10.5465/amr.2014.0384

[ref37] Delbecq A. House R. J. de Luque M. S. Quigley N. R. (2013). Implicit motives, leadership, and follower outcomes: an empirical test of CEOs. J. Leadersh. Organ. Stud. 20, 7–24. doi: 10.1177/1548051812467207

[ref38] Devloo T. Anseel F. De Beuckelaer A. (2011). Do managers use feedback seeking as a strategy to regulate demands–abilities misfit? The moderating role of implicit person theory. J. Bus. Psychol. 26, 453–465. doi: 10.1007/s10869-010-9200-7

[ref39] Dulebohn J. H. Bommer W. H. Liden R. C. Brouer R. L. Ferris G. R. (2012). A meta- analysis of antecedents and consequences of leader-member exchange: integrating the past with an eye toward the future. J. Manag. 38, 1715–1759. doi: 10.1177/0149206311415280

[ref40] Edwards J. R. (2002). “Alternatives to difference scores: polynomial regression analysis and response surface methodology” in Measuring and analyzing behavior in organizations: Advances in measurement and data analysis. eds. Drasgow F. Schmitt N. (San Francisco, CA: Jossey-Bass), 350–400.

[ref41] Edwards J. R. Cable D. M. (2009). The value of value congruence. J. Appl. Psychol. 94, 654–677. doi: 10.1037/a0014891, PMID: 19450005

[ref42] Edwards J. R. Cable D. M. Williamson I. O. Lambert L. S. Shipp A. J. (2006). The phenomenology of fit: linking the person and environment to the subjective experience of person-environment fit. J. Appl. Psychol. 91, 802–827. doi: 10.1037/0021-9010.91.4.802, PMID: 16834507

[ref43] Efron B. Tibshirani R. J. (1993). An introduction to the bootstrap. New York: Chapman & Hall.

[ref9001] El-Dabt L. AlReshaid F. Park K. AlBuloushi N. Al-Enzi A. (2025). Sustainable strategic nation branding through sports: leveraging soft power via mega-event hosting. Frontiers in Sociology 10, 1521396. doi: 10.3389/fsoc.2025.1521396 40134517 PMC11934112

[ref44] Emirza S. Katrinli A. (2022). Great minds think alike: does leader-follower similarity in construal level of the work enhance leader-member exchange quality? Leadersh. Organ. Dev. J. 43, 181–195. doi: 10.1108/LODJ-04-2021-0169

[ref45] Epitropaki O. Kapoutsis I. Ellen B. P. Ferris G. R. Drivas K. Ntotsi A. (2016). Navigating uneven terrain: the roles of political skill and LMX differentiation in prediction of work relationship quality and work outcomes. J. Organ. Behav. 37, 1078–1103. doi: 10.1002/job.2100

[ref46] Epitropaki O. Kark R. Mainemelis C. Lord R. G. (2017). Leadership and followership identity processes: a multilevel review. Leadersh. Q. 28, 104–129. doi: 10.1016/j.leaqua.2016.10.003

[ref47] Epitropaki O. Martin R. (2005). From ideal to real: a longitudinal study of implicit leadership theories, leadership member exchanges, and employee outcomes. J. Appl. Psychol. 90, 659–676. doi: 10.1037/0021-9010.90.4.659 16060785

[ref48] Epitropaki O. Sy T. Martin R. Tram-Quon S. Topakas A. (2013). Implicit leadership and followership theories “in the wild”: taking stock of information-processing approaches to leadership and followership in organizational settings. Leadersh. Q. 24, 858–881. doi: 10.1016/j.leaqua.2013.10.005

[ref49] Erdil G. E. Tanova C. (2015). Do birds of a feather communicate better? The cognitive style congruence between managers and their employees and communication satisfaction. Stud. Psychol. 57, 177–193. doi: 10.21909/sp.2015.03.692

[ref50] Erdogan B. Bauer T. N. (2014). “Leader-member exchange (LMX) theory: the relational approach to leadership” in The Oxford handbook of leadership and organizations. ed. Day D. V. (New York: Oxford University Press Inc), 407–433.

[ref51] Erkutlu H. (2008). The impact of transformational leadership on organizational and leadership effectiveness: the Turkish case. J. Manage. Dev. 27, 708–726. doi: 10.1108/02621710810883616

[ref52] Erturk A. (2008). A trust-based approach to promote employees’ openness to organizational change in Turkey. Int. J. Manpow. 29, 462–483. doi: 10.1108/01437720810888580

[ref53] Erturk A. Van den Broeck H. Verbrigghe J. (2018). Self-other agreement on supervisor’s transformational leadership and subordinates’ assessment of supervisor’s performance: mediating role of leader-member exchange. Leadersh. Organ. Dev. J. 39, 291–308. doi: 10.1108/LODJ-02-2016-0048

[ref54] Fleenor J. W. Smither J. Atwater L. Braddy P. Sturm R. (2010). Self-other rating agreement in leadership: a review. Leadersh. Q. 21, 1005–1034. doi: 10.1016/j.leaqua.2010.10.006

[ref55] Fornell C. Larcker D. (1981). Evaluating structural equation models with unobservable variables and measurement error. J. Mark. Res. 18, 39–50. doi: 10.2307/3151312

[ref56] Foti R. J. Hansbrough T. K. Epitropaki O. Coyle P. T. (2017). Dynamic viewpoints on implicit leadership and followership theories: approaches, findings, and future directions. Leadersh. Q. 28, 261–267. doi: 10.1016/j.leaqua.2017.02.004

[ref57] Gardner W. L. Cogliser C. C. Davis K. M. Dickens M. (2011). Authentic leadership theory and research: a review of the literature and research agenda. Leadersh. Q. 22, 1120–1145. doi: 10.1016/j.leaqua.2011.09.007

[ref58] Gooty J. Serban A. Thomas J. S. Gavin M. B. Yammarino F. J. (2012). Use and misuse of levels of analysis in leadership research: an illustrative review of leader-member exchange. Leadersh. Q. 23, 1080–1103. doi: 10.1016/j.leaqua.2012.10.002

[ref59] Gottfredson R. K. Aguinis H. (2017). Leadership behaviors and follower performance: deductive and inductive examination of theoretical rationales and underlying mechanisms. J. Organ. Behav. 38, 558–591. doi: 10.1002/job.2152

[ref60] Gottfredson R. K. Wright S. L. Heaphy E. D. (2020). A critique of the leader-member exchange construct: back to square one. Leadersh. Q. 31:101385. doi: 10.1016/j.leaqua.2020.101385

[ref61] Graen G. B. Uhl-Bien M. (1995). Relationship-based approach to leadership: development of leader-member exchange (LMX) theory of leadership over 25 years: applying a multi-level multi-domain perspective. Leadersh. Q. 6, 219–247. doi: 10.1016/1048-9843(95)90036-5

[ref62] Guillén L. Mayo M. Korotov K. (2015). Is leadership a part of me? A leader identity approach to understanding the motivation to lead. Leadersh. Q. 26, 802–820. doi: 10.1016/j.leaqua.2015.05.001

[ref63] Hair J. F. Black B. Babin B. Anderson R. E. Tatham R. L. (2006). Multivariate data analysis. 6th Edn. Upper Saddle River, NJ: Prentice-Hall.

[ref64] Haslam S. A. van Knippenberg D. Platow M. J. Ellemers N. (2015). Social identity at work: Developing theory for organizational practice. New York, NY: Psychology Press.

[ref65] Hejazi A. (2016). The relationship between managers’ cognitive style and their relationship type as moderated by organizational culture. Arab. J. Bus. Manag. Rev. 6, 242–252. doi: 10.4172/2223-5833.1000242

[ref66] Hirst G. Walumbwa F. Aryee S. Butarbutar I. Chen C. (2016). A multi-level investigation of authentic leadership as an antecedent of helping behavior. J. Bus. Ethics 139, 485–499. doi: 10.1007/s10551-015-2580-x

[ref67] Hogg M. A. Terry D. J. (2000). Social identity and self-categorization processes in organizational contexts. Acad. Manag. Rev. 25, 121–140. doi: 10.2307/259266

[ref68] Hu J. Liden R. C. (2013). Relative leader-member exchange within team contexts: how and when social comparison impacts individual effectiveness. Pers. Psychol. 66, 127–172. doi: 10.1111/peps.12008

[ref69] Jansen K. J. Kristof-Brown A. L. (2005). Marching to the beat of a different drummer: examining the impact of pacing congruence. Organ. Behav. Hum. Decis. Process. 97, 93–105. doi: 10.1016/j.obhdp.2005.03.005

[ref70] John O. P. Srivastava S. (1999). “The big five trait taxonomy: history, measurement, and theoretical perspectives” in Handbook of personality: Theory and research. eds. Pervin L. A. John O. P.. 2nd ed (New York: Guilford), 102–138.

[ref71] Johnson J. W. Ferstl K. L. (1999). The effects of interrater and self-other agreement on performance improvement following upward feedback. Pers. Psychol. 52, 271–303. doi: 10.1111/j.1744-6570.1999.tb00162.x

[ref72] Karakitapoğlu-Aygün Z. Gumusluoglu L. Erturk A. Scandura T. (2021). Two to tango? A cross-cultural investigation of the leader-follower agreement on authoritarian leadership. J. Bus. Res. 128, 473–485. doi: 10.1016/j.jbusres.2021.02.034

[ref73] Lee A. Carpenter N. C. (2018). Seeing eye to eye: a meta-analysis of self-other agreement of leadership. Leadersh. Q. 29, 253–275. doi: 10.1016/j.leaqua.2017.06.002, PMID: 40433219

[ref74] Lee A. Thomas G. Martin R. Guillaume Y. (2019). Leader-member exchange (LMX) ambivalence and task performance: the cross-domain buffering role of social support. J. Manage. 45, 1927–1957. doi: 10.1177/0149206317741190

[ref9002] Liden R. C. Maslyn J. M. (1998). Multidimensionality of leader–member exchange: An empirical assessment through scale development Journal of Management 24, 43–72. doi: 10.1016/S0149-2063(99)80053-1

[ref75] Lord R. G. Dinh J. E. (2014). What have we learned that is critical in understanding leadership perceptions and leader-performance relations? Ind. Organ. Psychol. 7, 158–177. doi: 10.1111/iops.12127

[ref76] Ludwig R. M. Srivastava S. Berkman E. T. (2019). Predicting exercise with a personality facet: Planfulness and goal achievement. Psychol. Sci. 30, 1510–1521. doi: 10.1177/0956797619868812, PMID: 31526324 PMC7197217

[ref77] Ludwig R. M. Srivastava S. Berkman E. T. Donnellan B. (2018). Planfulness: a process-focused construct of individual differences in goal achievement. Collabra Psychol. 4. doi: 10.1525/collabra.136

[ref78] Martin R. Guillaume Y. Thomas G. Lee A. Epitropaki O. (2016). Leader-member exchange (LMX) and performance: a meta-analytic review. Pers. Psychol. 69, 67–121. doi: 10.1111/peps.12100

[ref79] Matta F. K. Scott B. A. Koopman J. Conlon D. E. (2015). Does seeing “eye to eye” affect work engagement and organizational citizenship behavior? A role theory perspective on LMX agreement. Acad. Manag. J. 58, 1686–1708. doi: 10.5465/amj.2014.0106

[ref80] Matta F. K. Van Dyne L. (2020). Understanding the disparate behavioral consequences of LMX differentiation: the role of social comparison emotions. Acad. Manag. Rev. 45, 154–180. doi: 10.5465/amr.2016.0264

[ref81] Moharrak M. AlReshaid F. Park K. M. Alsaber A. R. (2025). International hidden entrepreneurs: concealed partnerships in new venture formation in an emerging markets context. J. Innov. Knowl. 10:100669. doi: 10.1016/j.jik.2025.100669

[ref82] Nahrgang J. D. Seo J. J. (2016). “How and why high leader-member exchange (LMX) relationships develop: examining the antecedents of LMX” in The Oxford handbook of leader-member exchange. eds. Bauer T. N. Erdogan B. (New York, USA: Oxford University Press), 87–118.

[ref83] Nielsen M. B. Eid J. Mearns K. Larsson G. (2013). Authentic leadership and its relationship with risk perception and safety climate. Leadersh. Organ. Dev. J. 34, 308–325. doi: 10.1108/LODJ-07-2011-0065

[ref84] Nunnally J. (1978). Psychometric methods. 2nd Edn. New York: McGraw-Hill.

[ref85] Ostroff C. Atwater L. Feinberg B. (2004). Understanding self– other agreement: a look at rater and ratee characteristics, context, and outcomes. Pers. Psychol. 57, 333–375. doi: 10.1111/j.1744-6570.2004.tb02494.x

[ref86] Piccolo R. F. Bono J. E. Heinitz K. Rowold J. Duehr E. Judge T. A. (2012). The relative impact of complementary leader behaviors: which matter most? Leadersh. Q. 23, 567–581. doi: 10.1016/j.leaqua.2011.12.008

[ref87] Podsakoff P. M. MacKenzie S. B. Lee J. Y. Podsakoff N. P. (2003). Common method biases in behavioral research: a critical review of the literature and recommended remedies. J. Appl. Psychol. 88, 879–903. doi: 10.1037/0021-9010.88.5.879, PMID: 14516251

[ref88] Podsakoff P. M. MacKenzie S. B. Moorman R. H. Fetter R. (1990). Transformational leadership behaviors and their effect on followers’ trust in leader, satisfaction and OCB’S. Leadersh. Q. 1, 107–142. doi: 10.1016/1048-9843(90)90009-7

[ref89] Richardson H. A. Simmering M. J. Sturman M. C. (2009). A tale of three perspectives: examining post hoc statistical techniques for detection and correction of common method variance. Organ. Res. Methods 12, 762–800. doi: 10.1177/1094428109332834

[ref90] Riggs B. S. Porter C. O. L. H. (2017). Are there advantages to seeing leadership the same? A test of the mediating effects of LMX on the relationship between ILT congruence and employees’ development. Leadersh. Q. 28, 285–299. doi: 10.1016/j.leaqua.2016.10.009

[ref91] Rockstuhl T. Dulebohn J. H. Ang S. Shore L. M. (2012). Leader–member exchange (LMX) and culture: a meta-analysis of correlates of LMX across 23 countries. J. Appl. Psychol. 97, 1097–1130. doi: 10.1037/a0029978, PMID: 22985117

[ref92] Sass D. A. Smith P. L. (2006). The effects of parceling unidimensional scales on structural equation modeling. Struct. Equ. Modeling 13, 566–586. doi: 10.1207/s15328007sem1304_4

[ref93] Sluss D. M. Ployhart R. E. Cobb M. G. Ashforth B. E. (2012). Generalizing newcomers' relational and organizational identifications: processes and prototypicality. Acad. Manag. J. 55, 949–975. doi: 10.5465/amj.2010.0420

[ref94] Smither J. W. London M. Reilly R. R. (2005). Does performance appraisal improve following multisource feedback? A theoretical model, meta-analysis, and review of empirical findings. Pers. Psychol. 58, 33–66. doi: 10.1111/j.1744-6570.2005.514_1.x

[ref95] Snijders T. A. B. Bosker R. J. (2012). *Multilevel* analysis: An introduction to basic and advanced multilevel modeling. London: Sage.

[ref96] Sosik J. J. (2001). Self-other agreement on charismatic leadership: relationships with work attitudes and managerial performance. Group Organ. Manag. 26, 484–511. doi: 10.1177/1059601101264005

[ref97] Sosik J. J. Godshalk V. M. (2004). Self-other rating agreement in mentoring group organ. Management 29, 442–469. doi: 10.1177/1059601103257421

[ref98] Sosik J. J. Megerian L. E. (1999). Understanding leader emotional intelligence and performance. Group Organ. Manag. 25, 291–317. doi: 10.1177/1059601199243006

[ref99] Sosik J. J. Potosky D. Jung D. I. (2002). Adaptive self-regulation: meeting others' expectations of leadership and performance. J. Soc. Psychol. 142, 211–232. doi: 10.1080/00224540209603896 11999873

[ref100] Steffens N. K. Haslam S. A. Reicher S. D. Platow M. J. Fransen K. Yang J.. (2014). Leadership as social identity management: introducing the identity leadership inventory (ILI) to assess and validate a four-dimensional model. Leadersh. Q. 25, 1001–1024. doi: 10.1016/j.leaqua.2014.05.002

[ref101] Steffens N. K. Munt K. A. van Knippenberg D. Platow M. J. Haslam S. A. (2021). Advancing the social identity theory of leadership: a meta-analytic review of leader group prototypicality. Organ. Psychol. Rev. 11, 35–72. doi: 10.1177/2041386620962569

[ref102] Sui Y. Wang H. Kirkman B. L. Li N. (2016). Understanding the curvilinear relationships between LMX differentiation and team coordination and performance. Pers. Psychol. 69, 559–597. doi: 10.1111/peps.12115

[ref103] Tekleab A. G. Sims H. P. Yun S. Tesluk P. E. Cox J. (2008). Are we on the same page? Effects of self-awareness of empowering and transformational leadership. J. Leadersh. Organ. Stud. 14, 185–201. doi: 10.1177/1071791907311069

[ref104] Tsai C. Y. Dionne S. D. Wang A. C. Spain S. M. Yammarino F. J. Cheng B. S. (2017). Effects of relational schema congruence on leader-member exchange. Leadersh. Q. 28, 268–284. doi: 10.1016/j.leaqua.2016.11.005

[ref105] Beurden J. Van de Voorde K. Veldhoven M.V. Jiang K. Do managers and employees see eye to eye? A dyadic perspective on high-performance work practices and their impact on performance J. Bus. Res. (2025). 190:115190 doi: 10.1016/j.jbusres.2025.115190

[ref106] Vanderheyden K. De Baets S. (2015). Does cognitive style diversity affect performance in dyadic student teams. Learn. Individ. Differ. 38, 143–150. doi: 10.1016/j.lindif.2015.01.006

[ref107] Vecchio R. P. Anderson R. J. (2009). Agreement in self-other ratings of leader effectiveness: the role of demographics and personality. Int. J. Sel. Assess. 17, 165–179. doi: 10.1111/j.1468-2389.2009.00460.x, PMID: 40421581

[ref108] Von Wittich D. Antonakis J. (2011). The KAI cognitive style inventory: was it personality all along? Pers. Individ. Differ. 50, 1044–1049. doi: 10.1016/j.paid.2011.01.022

[ref109] Walumbwa F. O. Avolio B. J. Gardner W. L. Wernsing T. S. Peterson S. J. (2008). Authentic leadership: development and validation of a theory-based measure. J. Manag. 34, 89–126. doi: 10.1177/0149206307308913

[ref110] Wang H. Law K. S. Hackett R. D. Wang D. Chen Z. X. (2005). Leader-member exchange as a mediator of the relationship between transformational leadership and followers’ performance and organizational citizenship behavior. Acad. Manag. J. 48, 420–432. doi: 10.5465/AMJ.2005.17407908

[ref111] Wang H. Sui Y. Luthans F. Wang D. Wu Y. (2014). Impact of authentic leadership on performance: role of followers’ positive psychological capital and relational processes. J. Organ. Behav. 35, 5–21. doi: 10.1002/job.1850

[ref112] Wayne S. J. Shore L. M. Liden R. C. (1997). Perceived organizational support and leader- member exchange: a social exchange perspective. Acad. Manag. J. 40, 82–111. doi: 10.2307/257021

[ref113] Yukl G. A. (2020). Leadership in organizations. 9th Edn. Boston, MA Pearson.

[ref114] Zhang L. F. Sternberg R. J. (2005). A threefold model of intellectual styles. Educ. Psychol. Rev. 17, 1–53. doi: 10.1007/s10648-005-1635-4

[ref115] Zhang Z. Wang M. Shi J. (2012). Leader-follower congruence in proactive personality and work outcomes: the mediating role of leader-member exchange. Acad. Manag. J. 55, 111–130. doi: 10.5465/amj.2009.0865

